# Development of Arthritis in a Large Real-World Cohort of Patients With Pediatric Onset Psoriasis

**DOI:** 10.1177/24755303261446205

**Published:** 2026-04-25

**Authors:** Malak Al-Gawahiri, Elke M. G. J. de Jong, Ellen J. H. Schatorjé, Esther P. A. H. Hoppenreijs, Juul M. P. A. van den Reek, Marieke M.B. Seyger

**Affiliations:** 1 Department of dermatology, 6034 Radboud University Medical Center, Nijmegen, The Netherlands; 2 Department of Pediatric Rheumatology and Immunology, Amalia Children’s Hospital, 6034 Radboud University Medical Center, Nijmegen, The Netherlands

**Keywords:** psoriasis, pediatric psoriasis, juvenile psoriatic arthritis, psoriatic arthritis, sex, obesity, nail psoriasis

## Abstract

**Background:**

Early identification and treatment of psoriatic arthritis (PsA) among patients with psoriasis is important to prevent joint damage. Insights in arthritis onset and the associated clinical factors among pediatric and young adult patients with psoriasis is scarce.

**Objectives:**

To describe pediatric patients with psoriasis who subsequently developed psoriatic arthritis at pediatric age or in young adulthood (JPsA/PsA). We focused on clinical features and timing of onset, and compared clinical features to characteristics of a large cohort of pediatric psoriasis patients.

**Methods:**

Data on patients with pediatric onset of psoriasis were obtained from the prospective, daily practice, ChildCAPTURE registry. Descriptive statistics were used. ILAR and CASPAR criteria were used for classification of JPsA and PsA, respectively. The time to JPsA/PsA diagnosis following psoriasis onset was analyzed using Kaplan-Meier survival analysis.

**Results:**

Among 717 pediatric and young adult patients with psoriasis, 15 (2.1%) developed arthritis, of which 8 patients before the age of 18 years (1.1%, JPsA) and 7 patients in young adulthood (1.0%, PsA), with an estimated incidence of 2.8% within 10 years after psoriasis diagnosis. The median interval between psoriasis onset and JPsA/PsA development was 4.8 years. Male sex, obesity, and nail involvement were common clinical features of patients developing JPsA/PsA, and these were observed more frequently compared to the total pediatric psoriasis cohort.

**Conclusion:**

The incidence of JPsA/PsA among patients with pediatric onset of psoriasis is low. If the clinical features male sex, obesity and nail involvement are present, extra awareness for the development of JPsA/PsA is warranted.

## Introduction

Early diagnosis of psoriatic arthritis (PsA) in patients with psoriasis is important for prevention and interception of PsA.^1^ Many potential risk factors for PsA development in adult patients with psoriasis have been suggested in the literature including obesity, nail involvement, psoriasis severity, familial history, and arthralgia.^1-4^ However, little is known about the development of PsA in pediatric patients with psoriasis.

At the moment, the largest body of evidence on the development of arthritis in pediatric patients with psoriasis originates from pediatric rheumatology cohorts, describing patients with Juvenile Psoriatic Arthritis (JPsA). JPsA is a subtype of Juvenile Idiopathic Arthritis (JIA) and accounts for approximately 8% (range 4-11%) of the JIA population.^5-9^ Despite its prevalence, diagnosing JPsA remains challenging, particularly in patients without psoriatic lesions (in 40-60% of JPsA cases^10,11^), due to the heterogeneous patient population characterized by diverse clinical presentations and laboratory findings.^5,12-14^ Consequently, the clinical features of these JPsA cohorts may not correspond to the clinical characteristics of pediatric patients with psoriasis subsequently developing JPsA, who are primarily seen in a dermatology setting.

Only one claims-based study reported on the development of JPsA in pediatric patients with psoriasis and its associated risk factors, finding that 2% of children with psoriasis developed JPsA during a mean follow-up of 5 years. Older age at the time of first psoriasis diagnosis and the presence of uveitis were identified as risk factors for developing JPsA.^10^ Especially in pediatric and young adult patients with psoriasis, little is known about the presenting clinical features of psoriatic arthritis and potential risk factors thereof. This study aims to describe a real-world cohort of pediatric patients with psoriasis who subsequently developed psoriatic arthritis at pediatric age or in young adulthood (JPsA/PsA), focusing on their clinical features and timing of onset. In addition, we compare these clinical features with the characteristics of pediatric psoriasis patients from the same cohort who did not develop psoriatic arthritis.

## Methods

This explorative study was conducted among pediatric patients with plaque psoriasis. Data were extracted from the ChildCAPTURE registry (Continuous Assessment of Psoriasis Treatment Use REgistry), a prospective, daily clinical practice registry in which pediatric patients with psoriasis (<18 years) treated at the Department of Dermatology at the Radboud University Medical Center in Nijmegen, the Netherlands, are included since 2008.^15^ All patients with dermatologist-confirmed psoriasis (ranging from mild to severe psoriasis) were included. Only those in whom the diagnosis could not be clearly established were excluded from the registry. Patients were referred by various general practitioners and dermatologists across the Netherlands to our dermatology department. Because pediatric patients included in the ChildCAPTURE registry are followed into young adulthood, data on psoriatic arthritis onset beyond the age of 18 years were also extracted. Follow-up duration varied between individuals, with data collection extending up to 30 years of age. This study was reviewed and approved by the local ethics committee. Written informed consent was obtained from the parents or guardians and/or from the participating pediatric patients according to applicable rules.

### Data collection

At the time of registry inclusion (baseline), data were extracted on patient characteristics, including sex, age at onset of psoriasis, psoriasis treatment history, family history of psoriasis and psoriatic arthritis. The following clinical characteristics were extracted: Psoriasis Area and Severity Index (PASI) score, Body Surface Area (BSA), Body Mass Index (BMI), and nail involvement. At each follow-up visit, the following data were recorded: PASI score, BSA, BMI, and nail involvement. The age at onset of JPsA (defined as PsA <18 years of age) or PsA (defined as PsA ≥18 years of age) was recorded,^16^ either at baseline if already diagnosed, or during follow-up if the diagnosis occurred later. The total group was designated as JPSA/PsA.

For BMI, children (<18 years) were classified as underweight, normal weight, overweight or obese according to International Obesity Task Force criteria.^17,18^ For young adults (>18 years), BMI categories were defined according to the World Health Organization reference ranges.^19^ Nail involvement was defined as the presence of nail psoriasis features such as pitting, onycholysis or oil drop phenomenon.

### Diagnosis of JPsA/PsA

Patients were referred to a rheumatologist based on clinical assessment by the treating physician. Factors considered for referral included persistent joint complaints, morning stiffness lasting more than 30 minutes, and elevated PEST scores (>3). Patients were referred to a (pediatric) rheumatologist at the patient’s preferred hospital, either at the Radboud University Medical Center or another medical center of their choice. Medical records from these evaluations at the time of the arthritis diagnosis, as well as laboratory results and imaging studies, were obtained retrospectively for this study. Children <18 years were diagnosed with JPsA by a pediatric rheumatologist according to the ILAR classification criteria.^16,20,21^The ILAR criteria define JPsA as psoriasis and arthritis, or arthritis without psoriasis and at least two of the following: (1) dactylitis, (2) nail pitting or onycholysis, (3) psoriasis in a first-degree relative.^21^

Young adults (≥ 18 years) were diagnosed with PsA by a rheumatologist according to the CASPAR criteria.^22^ The CASPAR criteria require the presence of inflammatory articular disease (affecting joints, spine, or entheses) along with a score of ≥3 points from the following categories: (1) current psoriasis, personal or family history of psoriasis; (2) typical psoriatic nail dystrophy; (3) negative rheumatoid factor; (4) current or past dactylitis; and (5) radiographic evidence of juxta articular new bone formation.^22^

### Statistical Analysis

Analyses were performed in SPSS version 29.0 (IBM Corporation, Armonk, NY, USA). Descriptive statistics were used to summarize patient characteristics, including demographics and clinical features. Categorical variables were presented as numbers and percentages, and continuous variables as medians with interquartile ranges [IQRs] or means with standard deviation (SD), depending on data distribution. Baseline characteristics between patients with JPsA/PsA and the total cohort were compared descriptively; numbers of patients with JPsA/PsA were low therefore statistical comparisons were not made. The time to arthritis diagnosis following psoriasis onset was analyzed using Kaplan-Meier survival analysis in R studio version 2024.04 (R Foundation for Statistical Computing, Vienna, Austria). Here, JPsA/PsA diagnosis was considered an event, and patients that were lost-to-follow up or patients who remained arthritis-free until data lock were censored.

## Results

A total of 717 pediatric patients with psoriasis were included in the study, with a cumulative observation period of 2850 years following inclusion in the ChildCAPTURE registry. The majority of patients were female (60.1%) and the median age at psoriasis diagnosis was 8.0 [6.2] years (Table 1). Of 717 patients, 15 patients (2.1%) developed JPsA/PsA. Over a 10-year period following psoriasis onset, the cumulative incidence of development of JPsA/PsA was 2.8% (95%CI = 1.0%-4.6%) as shown in the Kaplan-Meier analysis in Supplemental Figure 1.

**Table 1.** Clinical Characteristics of Pediatric Patients in the ChildCAPTURE Registry

	All patients (n = 717)	Patients diagnosed with JPsA/PsA (n = 15)
Sex, n (%)		
Male	286 (39.9)	10 (66.7)
Female	431 (60.1)	5 (33.3)
Age at psoriasis diagnosis, years, median [IQR]	8.0 [6.2]	12.0 [7.4]
Age at registry inclusion, years, median [IQR]	11.2 [6.8]	14.6 [4.4]
Age at arthritis diagnosis, years, median [IQR]	—	17.3 [9.6]
Follow-up duration in registry, years, median [IQR]^a^	2.8 [5.6]	9.7 [7.6]
Time from psoriasis onset to arthritis diagnosis, years, median [IQR]	—	4.8 [7.5]
First degree family history, n (%)^a^		
Psoriasis	228 (31.8)	5 (33.3)
Psoriatic arthritis	19 (3.8)	0 (0.0)
BMI category at registry inclusion, n (%)^b^		
Underweight	86 (15.6)	0 (0.0)
Normal weight	361 (65.4)	8 (61.5)
Overweight	68 (12.3)	1 (7.7)
Obese	37 (6.7)	4 (30.8)
BMI category at arthritis diagnosis, n (%)^a,b^		
Underweight		0 (0.0)
Normal weight	—	5 (41.7)
Overweight		4 (33.3)
Obese		3 (25.0)
Nail involvement at registry inclusion, n (%)^a^	129 (18.0)	6 (40.0)
Nail involvement at arthritis diagnosis, n (%)^a^	—	7 (50.0)
Disease severity at registry inclusion, median [IQR]		
PASI (0-72)	5.6 [4.8]	6.3 [2.7]
BSA (0-100%)	6.0 [8.0]	6.6 [7.0]
Disease severity at arthritis diagnosis, median [IQR]^a^		
PASI (0-72)	—	3.9 [7.6]
BSA (0-100%)		2.5 [6.9]

*Notes.* Percentages were calculated using the total number of available data for that characteristic per group.

Abbreviations: BSA, Body surface area; CDLQI, Children’s Dermatology Life Quality Index; DLQI, Dermatology Life Quality Index; JPsA/PsA, juvenile psoriatic arthritis or psoriatic arthritis; PASI, Psoriasis Area and Severity Index; PEST, Psoriasis Epidemiology Screening Tool; PGA, Physician Global Assessment; PsA, psoriatic arthritis.

^a^Variables with *missing* data include: family history of psoriasis (all patients, n = 1), family history of psoriatic arthritis (all patients, n = 220; patients diagnosed with JPsA/PsA, n = 7), BMI category at registry inclusion (all patients, n = 165; patients diagnosed with JPsA/PsA, n = 2), BMI category at arthritis diagnosis (n = 3), nail involvement at registry inclusion (all patients, n = 1), nail involvement at arthritis diagnosis (n = 1), PASI at arthritis diagnosis (n = 3), BSA at registry inclusion (all patients, n = 11), BSA at JPsA/PsA diagnosis (n = 3).

^b^Children (<18 years) were classified as underweight, normal weight, overweight or obese according to International Obesity Task Force criteria. These criteria are based on pooled BMI data from six countries (including the Netherlands) and provide internationally recognized cut-off points for BMI categorization in children.^17,18^For young adults (>18 years), BMI categories were defined according to the World Health Organization reference ranges: underweight (<18.5), normal weight (18.5-24.9), overweight (≥25.0), and obese (≥30.0).^19^

**Figure 1.**
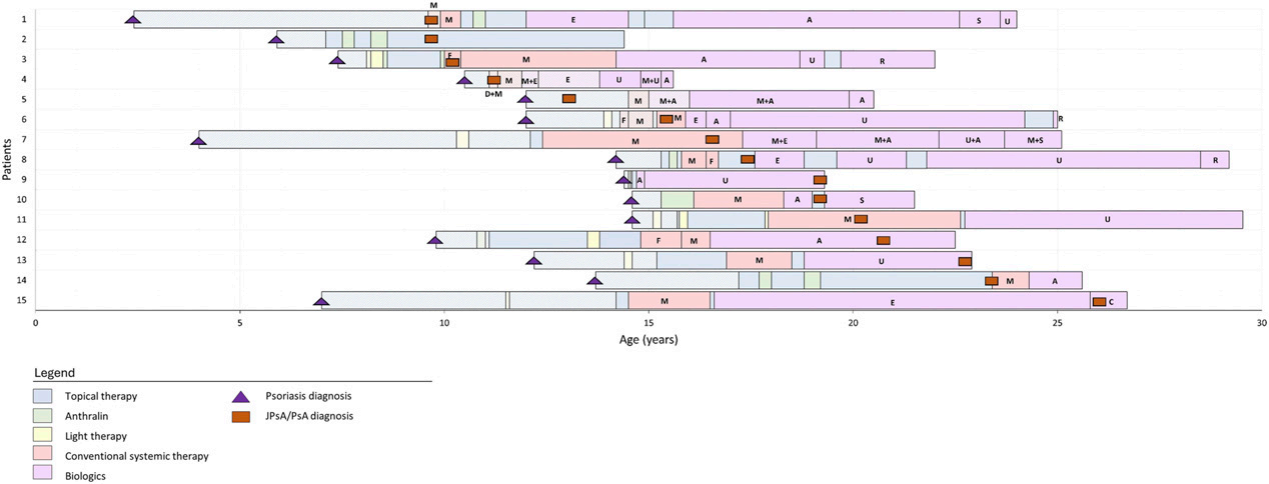
Psoriasis and JPsA/PsA onset and treatments of 15 pediatric and young adult patients with arthritis Abbreviations: JPsA/PsA, juvenile psoriatic arthritis or psoriatic arthritis; Ci, ciclosporin; M, methotrexate; F, fumaric acid A, adalimumab; C, certolizumab; E, etanercept; S, secukinumab; R, rituximab; U, ustekinumab

Among the 15 patients that developed JPsA/PsA, the median age at psoriasis diagnosis was 12.0 [7.4] years and the age at arthritis diagnosis was 17.3 [9.6] years (Table 1). The median time between onset of psoriasis and development of JPSA/PsA was 4.8 [7.5] years. Eight patients developed JPsA before the age of 18 years. Among all patients with JPsA/PsA, half of them had nail involvement and 58.3% were overweight or obese at the time of arthritis diagnosis. Furthermore, PASI and BSA were lower at time of JPsA/PsA diagnosis than at time of registry inclusion (PASI, 3.9 [7.6] vs 6.3 [2.7]; BSA, 2.5 [6.9] vs 6.6 [7.0]). At the time of JPsA/PsA diagnosis, 5 patients used topical therapy only, six were treated with conventional systemic therapy (methotrexate (n = 5) and fumaric acid (n = 1)), and four patients were treated with a biologic (adalimumab (n = 1), ustekinumab (n = 2) and certolizumab (n = 1)). More details on the treatments of these patients before and after diagnosis of JPsA/PsA are depicted in Figure 1.

A detailed rheumatological assessment at the time of JPsA/PsA diagnosis is described in Table 2. The age at psoriasis diagnosis ranged from 2.4 years to 14.6 years and diagnosis of JPsA/PsA ranged from 9.8 years to 26 years. The most common clinical findings at the time of JPsA/PsA diagnosis were asymmetry of the affected joints (9/12 patients) and involvement of the nails (7/14 patients). Oligoarthritis and polyarthritis were equally present in this group (7/13 vs 6/13 patients). Only 2 patients reported dactylitis, diagnosed based on clinical symptoms. Symptoms of enthesitis were not observed, and lower back pain was only observed in one patient, who was followed up with MRI and X-ray by the rheumatologist and revealed sacroiliitis and arthritis of the hip joints.

**Table 2.** Clinical and Laboratory Findings by (Pediatric) Rheumatologists in Pediatric and Young Adult Psoriasis Patients at Time of JPsA/PsA Diagnosis

Patient	Sex	Age at psoriasis diagnosis, years	Age at JPsA/PsA diagnosis, years	Arthritis	Dactylitis	Nail involvement	Enthesitis	Oligo- or polyarthritis	Asymmetry	Involvement of lower back	Change in therapy	Lab results					Erosions on imaging
												ANA	HLA-B27	CRP or ESR^a^	RF	Anti-CCP	
1	Female	2.4	9.8	Yes	No	No	No	Poly	Yes	No	Already on methotrexate, start ibuprofen	NEG	POS	Elevated	NEG	NEG	n.a.
2	Male	5.9	9.8	Yes	No	Yes	No	Oligo	Yes	No	Start naproxen	NEG	n.a.	n.a.	NEG	NEG	n.a.
3	Male	7.4	10,2	Yes	Yes	Yes	No	Poly	Yes	No	Already on fumaric acid, start naproxen	NEG	n.a.	Not elevated	NEG	NEG	Yes
4	Male	10.5	11.2	Yes	No	Yes	No	Poly	n.a.	No	Already on methotrexate	NEG	NEG	n.a.	NEG	n.a.	No
5	Male	12.0	13.0	Yes	No	Yes	No	Poly	No	No	Start naproxen	NEG	NEG	Not elevated	NEG	NEG	No
6	Female	14.6	15.5	Yes	n.a.	n.a.	n.a.	n.a.	n.a.	n.a.	Already on methotrexate	n.a.	n.a.	n.a.	n.a.	n.a.	n.a.
7	Male	4.0	16.5	Yes	No	No	No	Oligo	No	Yes	Already on methotrexate, start diclofenac	NEG	NEG	Not elevated	NEG	NEG	No
8	Male	14.2	17.3	Yes	n.a.	No	n.a.	n.a.	n.a.	n.a.	Initially start meloxicam, later enbrel	n.a.	n.a.	n.a.	n.a.	n.a.	n.a.
9	Female	14.4	19.3	Yes	Yes	No	No	Oligo	Yes	No	Already on ustekinumab, start sulfasalazine	POS	NEG	Elevated	NEG	NEG	No
10	Male	14.6	19.3	Yes	No	No	No	Poly	No	No	Start naproxen	n.a.	n.a.	Elevated	NEG	NEG	Yes
11	Female	14.6	20.2	Yes	No	Yes	No	Oligo	Yes	No	Injection kenacort, start naproxen and higher dosage methotrexate	n.a.	n.a.	n.a.	n.a.	n.a.	n.a.
12	Male	9.8	20,8	Yes	No	No	No	Poly	Yes	No	Already on adalimumab	NEG	n.a.	Not elevated	n.a.	NEG	No
13	Male	12.2	22.8	Yes	No	Yes	No	Oligo	Yes	No	Already on ustekinumab, injection kenacort	n.a.	n.a.	n.a.	n.a.	n.a.	n.a.
14	Male	13.7	23.4	Yes	No	No	No	Oligo	Yes	No	Start methotrexate	n.a.	n.a.	n.a.	n.a.	n.a.	n.a.
15	Female	7.0	26.0	Yes	No	Yes	No	Oligo	Yes	No	Already on certolizumab, start naproxen and injection depomedrol	n.a.	NEG	Not elevated	NEG	NEG	No

Abbreviations: n.a., not available; NEG, negative; POS, positive; CRP, C-reactive protein; ESR, erythrocyte sedimentation rate; ANA, antinuclear antibodies; HLA-B27, human leukocyte antigen B27; RF, rheumatoid factor; anti-CCP, anti-cyclic citrullinated peptide antibodies; DIP, distal interphalangeal joint; PIP, proximal interphalangeal joint.

^a^To assess whether CRP and ESR were elevated, the interpretation of the (pediatric) rheumatologist was used when available. If no interpretation was provided, CRP was considered elevated if ≥10 mg/L, and ESR was considered elevated if ≥15 mm/hour.

### Comparison Between Baseline Characteristics of JPsA/PsA Patients and the Total Pediatric Psoriasis Cohort

When comparing baseline characteristics between JPsA/PsA patients and the total pediatric psoriasis cohort (Table 1), it was observed that patients with JPsA/PsA were more often male (66.7% vs 39.9%), were more often obese (30.8% vs 6.7%) and their nails were more often affected at registry inclusion compared to the total psoriasis cohort (40.0% vs 18.0%). Also, the median age at psoriasis diagnosis is relatively higher (12 vs 8 years). Patients with JPsA/PsA had a longer median follow-up duration in the registry compared to the total cohort (9.7 [7.6] years vs 2.8 [5.6] years). Family history for psoriasis and PsA was comparable between groups.

## Discussion

This prospective registry study investigated the development of juvenile psoriatic arthritis and psoriatic arthritis (JPsA/PsA) among a large real-world cohort of patients with pediatric onset of psoriasis. It was shown that 2.1% of patients developed JPsA/PsA in this cohort. Age of arthritis onset was before the age of 18 years in eight patients (1.1%) and in young adulthood in seven patients (1.0%). The estimated cumulative risk of the development of JPsA/PsA was 2.8% within 10 years after the diagnosis of pediatric onset psoriasis. The median interval between psoriasis onset and JPsA/PsA development was 4.8 years. Patients with JPsA/PsA were predominantly male, more often obese, and had more often nail involvement compared to the total cohort.

In our cohort, with detailed characterization and follow-up, the pediatric patients with psoriasis that developed JPsA was 1.1%. Only one US based study using administrative claims data of children (0-16 years) identified that 2% of pediatric patients had diagnosis codes for both psoriasis and JPsA.^10^ However, after excluding the patients where the JPsA diagnosis preceded the psoriasis diagnosis, only 1.6% (70/4312) of pediatric patients with psoriasis developed JPsA.^10^ Our data confirm the fact that the percentage of pediatric patients with psoriasis that develops JPsA at pediatric age over time is low. In our study, we uniquely followed pediatric patients with psoriasis into young adulthood and showed that an additional 1.0% of patients developed PsA between the ages of 18 and 30 years.

In the present study, a median time from psoriasis to JPsA/PsA diagnosis of 4.8 years was found. Until now, only two studies mentioned the time from psoriasis diagnosis to development of arthritis in pediatric patients. In a US data claims study, the median time to index code for arthritis after index code for psoriasis was reported to be 17.6 months [IQR 4.1– 38.1],^10^ and in a retrospective case-control study among 14 pediatric patients, the mean time between psoriasis onset and arthritis diagnosis was 3.8 (± 3.5) years.^23^ While the time from psoriasis to JPsA/PsA diagnosis in our cohort is somewhat longer than previous mentioned pediatric reports, it remains considerably shorter than the 9-13 years that have been reported in adult cohorts.^23-25^ Although it is tentative to speculate that arthritis tends to occur relatively early in pediatric patients with psoriasis, this conclusion cannot be drawn with certainty due to the relative short follow-up and small number of patients.

Within our group of pediatric patients with psoriasis, the proportion of males was notably higher in the group that developed JPsA/PsA compared to the overall psoriasis group (66.7% vs 39.9%). In JPsA literature two distinct clinical subgroups with a bimodal age distribution for arthritis onset are identified, with incidence peaks occurring at approximately 2-3 years and 10-12 years of age.^12,24-26^ The later-onset peak more often presents with features characteristic of spondyloarthritis, including male predominance, enthesitis, axial involvement, and HLA-B27 positivity.^25^ The male predominance and later onset of arthritis found in our cohort supports this finding, although only one of our patients had axial involvement, HLA-B27 positivity was only found in one female patient and enthesitis was not recorded in any of our patients. These differences in clinical characteristics between JPsA cohorts derived from pediatric rheumatology practices and our dermatology derived cohort underline the importance of publications of pediatric cohorts in which psoriasis precedes arthritis.

In adults with psoriasis, obesity is the most significant modifiable risk factor for the development of PsA, as many studies have shown a relationship between high BMI and development of PsA.^2,27,28^ As there are limited studies focusing on pediatric patients with psoriasis who develop JPsA, the potential risk factors for PsA in the pediatric population remain poorly defined. However, a recent cross-sectional study identified higher BMI as possible predictor for the development of JPsA in children with psoriasis.^29^ In our cohort, 38.5% of patients that developed JPsA/PsA over time were overweight or obese at registry inclusion compared to 19.0% of the total cohort. At the time of JPsA/PsA diagnosis, 58.3% of patients was overweight or obese. Our data show a potential association between higher BMI and arthritis, and underscore previous literature in which a higher BMI was found to be a possible predictor for the development of JPsA in children with psoriasis.^29^

Interestingly, at the time of JPsA/PsA diagnosis, 6 of 15 patients were treated with conventional systemic therapy (methotrexate (n = 5) and fumaric acid (n = 1)), and four patients were on a biologic, including adalimumab (n = 1), ustekinumab (n = 2) and certolizumab (n = 1). Despite this, they still developed JPsA/PsA. We also observed that psoriasis severity was lower around the time of JPsA/PsA diagnosis compared to the time of registry inclusion for these 15 patients (PASI, 3.9 vs 6.3; BSA, 2.5 vs 6.6). Both findings suggest that JPsA/PsA can arise in pediatric and young adult patients when the psoriasis is clinically well-controlled.

Nail involvement has often been reported as a potential risk factor for PsA in adults, although findings across studies are somewhat inconsistent.^30-32^ While several adult cohorts have shown an increased risk of PsA in patients with nail changes, particularly nail pitting and onycholysis, others have not found a statistically robust association. This variability may reflect differences in how the presence of nail involvement is assessed. One pediatric study has likewise noted nail involvement as a possible predictor for JPsA development, although it is unclear whether the analysis accounted for confounding factors.^29^ In our cohort, patients diagnosed with JPsA/PsA had a higher prevalence of nail involvement at the time of registry inclusion compared to the total population (40.0% resp. 18.0%).

One of the strengths of this study is the use of the longstanding, prospective ChildCAPTURE registry. The prospective design enabled detailed follow-up of disease progression and tracking of clinical features. A limitation of this study is the relatively low number of JPsA/PsA development events, which restricts the study’s statistical power and may affect the robustness of the findings. To identify patients with JPsA/PsA, joint complaints and PEST scores were prospectively collected in the ChildCAPTURE registry, and patients were actively referred to a rheumatologist with persistent joint complaints, morning stiffness >30 min and/or PEST scores >3. Nevertheless, the ChildCAPTURE registry was not solely designed to identify patients who develop arthritis, which could theoretically have resulted in an underreporting of the number of patients with arthritis.

Another limitation of this study is that, despite the use of a longstanding registry cohort with a substantial number of observation years (2850 years), the number of patients who may still develop arthritis remains uncertain. This is reflected by the shorter median follow-up for the entire cohort (2.8 years) compared with those who have developed JPsA/PsA (9.7 years). To mitigate this, we performed Kaplan Meier analysis taking differences in follow-up duration into account.

In this study, a unique real-world cohort of patients with pediatric onset of psoriasis who subsequently developed JPsA/PsA was described. Approximately 1% of patients developed JPsA before the age of 18 years and another 1% during young adulthood, with an estimated incidence of 2.8% within 10 years after psoriasis diagnosis. The median interval between psoriasis onset and JPsA/PsA development was 4.8 years, and frequently found clinical features of patients developing arthritis included male sex, obesity, and nail involvement. These findings may help the early recognition of JPsA/PsA in pediatric and young adult patients with psoriasis and contribute to the ongoing efforts to refine classification criteria for juvenile psoriatic arthritis.

## Supplemental Material

**Supplemental Material -** Development of Arthritis in a Large Real-World Cohort of Patients With Pediatric Onset Psoriasis Supplemental Material for Development of Arthritis in a Large Real-World Cohort of Patients With Pediatric Onset Psoriasis by Malak Al-Gawahiri, Elke M. G. J. de Jong, Ellen J. H. Schatorjé, Esther P. A. H. Hoppenreijs, Juul M. P. A. van den Reek, Marieke M.B. Seyger in Journal of Psoriasis and Psoriatic Arthritis®

## Data Availability

Data are available upon request.*
