# Supplementary material for: Development of Arthritis in a Large Real-World Cohort of Patients With Pediatric Onset Psoriasis
Source: J Psoriasis Psoriatic Arthritis. 2026 Apr 25:24755303261446205. Online ahead of print. doi: 10.1177/24755303261446205 (PMC13110248; doi:10.1177/24755303261446205)
Supplement: Supplemental Material - Development of Arthritis in a Large Real-World Cohort of Patients With Pediatric Onset Psoriasis [file sj-pdf-1-jps-10.1177_24755303261446205.pdf]

**Supplemental material**

Supplemental material belonging to the publication: ‘Development of arthritis in a large real-world cohort of patients with pediatric onset psoriasis’, published in the *Journal of Psoriasis and Psoriatic Arthritis*, 2026.

**Supplemental figure 1.** Kaplan-Meier curve of time to JPsA-PsA development in pediatric and young adult patients with psoriasis[a]

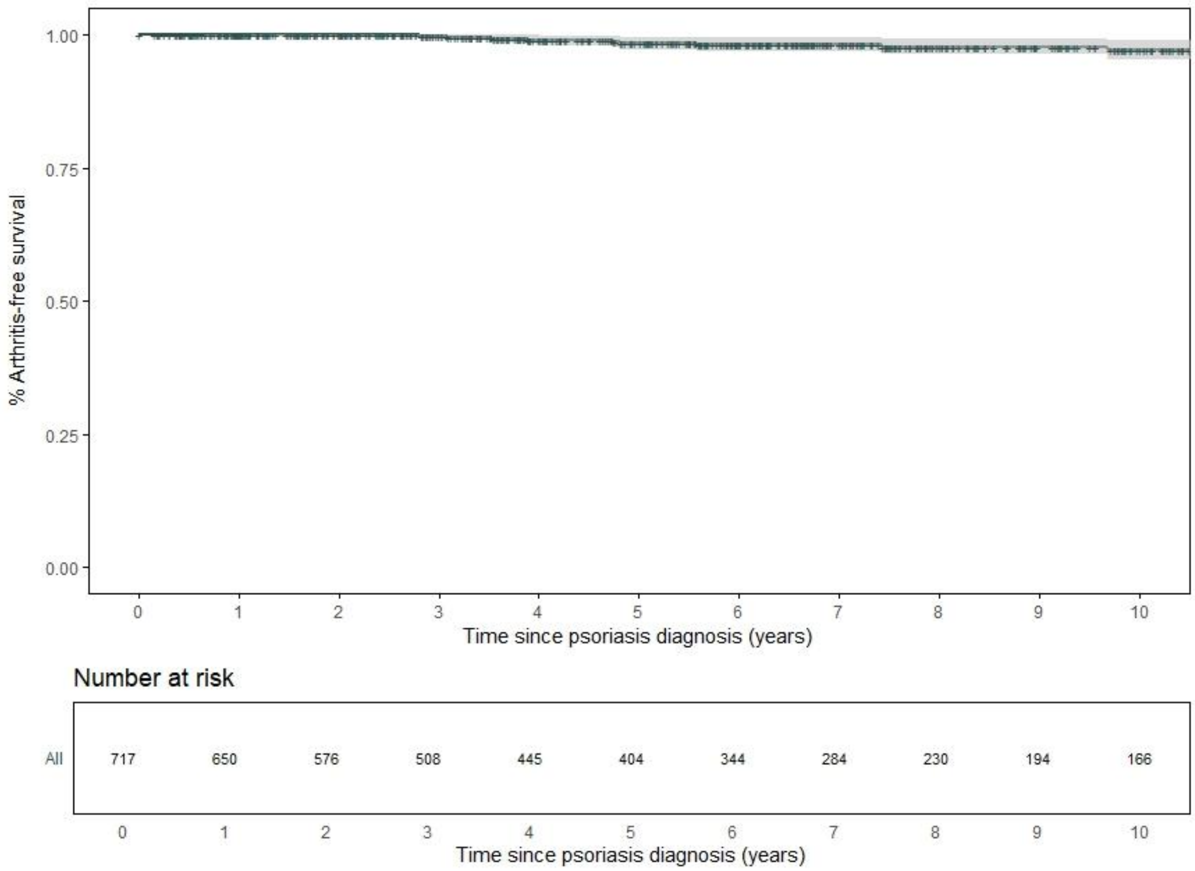


Abbreviation: JPsA/PsA, juvenile psoriatic arthritis or psoriatic arthritis

[a]Using Kaplan-Meier survival analysis in R, the 10-year cumulative incidence of JPsA/PsA development after psoriasis diagnosis was estimated at 2.8% (95% CI: 1.0%–4.6%).
